# Circulating levels of IL-33 are elevated by obesity and positively correlated with metabolic disorders in Chinese adults

**DOI:** 10.1186/s12967-021-02711-x

**Published:** 2021-02-04

**Authors:** Haoneng Tang, Ning Liu, Xiaojing Feng, Yanyi Yang, Yiyuan Fang, Siqi Zhuang, Yufeng Dai, Meilian Liu, Lingli Tang

**Affiliations:** 1 Department of Laboratory Medicine, The Second Xiangya Hospital, Central South University, Changsha, 410011 China; 2 Medical College, Yueyang Vocational and Technical College, Yueyang, 414000 China; 3 Health Management Center, The Second Xiangya Hospital, Central South University, Changsha, 410011 China; 4 Department of Biochemistry and Molecular Biology, University of New Mexico Health Sciences Center, Albuquerque, NM USA; 5 Department of Metabolism and Endocrinology, Metabolic Syndrome Research Center, Key Laboratory of Diabetes Immunology, Ministry of Education, National Clinical Research Center for Metabolic Diseases, The Second Xiangya Hospital, Central South University, Changsha, 410011 China

**Keywords:** IL-33, Obesity, Metabolic phenotype, Metabolic unhealthy overweight/obese, Metabolic disorders

## Abstract

**Background:**

Interleukin-33 (IL-33) plays a pivotal role in regulating innate immune response and metabolic homeostasis. However, whether its circulating level is correlated with obesity and metabolic disorders in humans remains largely unknown. We aimed to address this gap by determining IL-33 serum level and its downstream type 2 inflammatory cytokines interleukin-5 (IL-5) and interleukin-13 (IL-13) in overweight/obese population, and analyzing the specific associations between IL-33 and obesity metabolic phenotypes.

**Methods:**

217 subjects were enrolled and divided into three groups: healthy control (HC) subjects, metabolically healthy overweight/obese (MHOO) subjects and metabolically unhealthy overweight/obese (MUOO) subjects. Circulating levels of IL-33, IL-5 and IL-13 were measured using ELISA analyses. Multivariate regression analyses were further performed to determine the independent association between IL-33 and obesity metabolic phenotypes.

**Results:**

Circulating levels of IL-33 were significantly elevated in subjects of MUOO group compared with HC group and MHOO group, while no significant difference was observed between the latter two groups in IL-33 levels. Consistent with this, serum levels of IL-5/13 were higher in the MUOO group compared with HC and MHOO groups. After adjusted for all confounders, MUOO phenotype was significantly associated with increased IL-33 serum levels (OR = 1.70; 95% CI 1.09–2.64; *p* = 0.019). With the MHOO group as the reference population, higher circulating level of IL-33 was also positively associated with MUOO phenotype after adjusting for confounders (OR = 1.50; 95% CI 1.20–1.88; *p *= 2.91E−4). However, there was no significant association between MHOO phenotype and IL-33 levels (*p* = 0.942). Trend analysis further confirmed the positive correlation between MUOO phenotype and IL-33 level (*p* for trend = 0.019). Additionally, IL-33 was significantly and positively correlated with diastolic blood pressure (DBP), total cholesterol (TC), alanine aminotransferase (ALT), aspartate aminotransferase (AST), white blood cell (WBC), neutrophil and IL-5 only in MUOO group, while inversely correlated with high density lipoprotein cholesterol (HDL-C) in MHOO subjects.

**Conclusion:**

Circulating levels of IL-33 were significantly elevated in overweight/obese Chinese adults with metabolic disorders. Increased levels of IL-33 were positively associated with metabolically unhealthy overweight/obese phenotype and several metabolic syndrome risk factors.

## Introduction

Obesity and its related metabolic complications have become important public health issues globally, contributing to disease burden worldwide [1–4]. According to the recent research by WHO in 2017, more than two billion of people on the world are overweight and about one-third of them are obese [4]. With the occurrence and development of obesity, inflammatory immune cells infiltrated into adipose tissue and other metabolically active organs, causing inflammation and insulin resistance [5, 6]. Therefore, obesity has been well documented as a low-grade but chronic inflammatory disease [6–8], which contributes to the development and progression of its related metabolic complications such as cardiovascular diseases and type 2 diabetes mellitus (T2DM) [2]. However, the precise mechanism underlying obesity-induced inflammation remains incompletely understood.

IL-33, a newly discovered cytokine in the interleukin-1 (IL-1) family, is associated with Th1 and Th2 immune responses, which has been shown to be broadly expressed in various tissues and play a significant role as an ‘alarmin’ of some diseases [9–12]. Several reports have shown that IL-33 is important for maintaining immune cell homeostasis in adipose tissue [6] as well as the involvement of adipose tissue microenvironment in the obesity-related inflammation and complications [13]. In addition, IL-33 promotes the beiging of white adipocytes and energy expenditure by activating group 2 innate lymphoid cells (ILC2s) in adipose tissue, contracting obesity and its related disorders [14–16]. Despite the beneficial effects as an anti-inflammatory factor [9–11, 17], the double-edged sword effect of IL-33 in immunity and metabolism has gained growing attention [10, 11, 18]. Meanwhile, studies have also reported that IL-33 may play an undetermined role in obesity and its cardiovascular or diabetic complications since the specific role of IL-33 in the pathogenesis and development of several metabolic diseases seems to be inconsistent [9, 14–17]. Compared with its decoy receptor soluble ST2, IL-33 appears to be more complex [9, 18] and may have pro-inflammatory or anti-inflammatory effects in different environments [9, 18]. IL-33 expression was shown to be up-regulated by obesity in white adipose tissue, but resident ILC2s were paradoxically decreased in mice and humans [13, 14, 19]. Therefore, it is urgently needed to clarify whether systemic level and tissue level of IL-33 is differentially regulated by obesity.

Obese patients are mostly accompanied by a cluster of metabolic abnormalities such as hyperlipemia, hyperglycemia and hypertension [7], manifesting as metabolic syndrome [20, 21]. During the development of obesity, even at the overweight stage (BMI 25–28 kg/m^2^), some patients may have significant changes in their metabolic indicators with the disrupted metabolic immune balance [4, 7, 22, 23]. Thus, early intervention in overweight and obese people can effectively slow down the development of T2DM and cardiovascular events [22, 23]. Studies have indicated that two major types of overweight/obesity metabolic phenotypes have significant differences in inflammation levels [7, 24]. Metabolic healthy overweight/obesity (MHOO) phenotype is associated with a lower level of systemic inflammation [25]. However, when obesity progresses into metabolic unhealthy overweight/obesity (MUOO) phenotype, a higher inflammatory state occurs and is associated with T2DM and cardiovascular diseases [20, 26]. Therefore, understanding whether inflammation and metabolic syndrome risk factors contribute to IL-33 production during obesity progression will provide important evidence for further clarification of the interrelationship between obesity, metabolic health and inflammation [7].

Herein, we analyzed serum levels of IL-33 and its downstream Th2 cytokines IL-5/13 in overweight/obese population. We found a significant association between IL-33 circulating levels and MUOO phenotype. In addition, IL-33 was positively correlated with metabolic syndrome risk factors such as dyslipidemia and hypertension rather than obesity itself.

## Materials and methods

### Subjects

Initially, five hundred participants aged from 18 to 78 years were recruited from physical examination population in Second Xiangya Hospital of Central South University in China from Sep 2018 to Apr 2019. The exclusion criteria included cancer, organic heart disease, autoimmune disease, chronic hepatic or renal disease, hyperthyroidism, Cushing syndrome, hyperparathyroidism, infectious disease, pregnancy, lactation and surgery in the previous month. Additionally, participants who had recently (within 1 month) taken antihypertensive drugs, hypoglycemic drugs and antilipemic drugs had also been excluded. In order to compare the IL-33 serum levels in healthy population and overweight/obese population with or without metabolic abnormalities, a total sample size of 186 individuals was estimated by PASS15 software, since the two-sided test was required, with α of 0.05 and the power of 90%. Taken into account that 15% of patients may be lost during the follow-up, a total of 217 participants were eventually included in this study. Through matching, 144 overweight/obese subjects (BMI ≥ 25.0 kg/m^2^) were divided into the MHOO group (72 subjects) and MUOO group (72 subjects) according to their metabolic abnormalities, and 73 healthy participants were matched with both the MUOO and MHOO groups for gender and age. The study was conducted according to the guidelines of the Declaration of Helsinki and the study protocol was approved by the ethics committee of the Second Xiangya Hospital of Central South University (Ethics Number: 089/2016). Written informed consents were obtained from all participants.

### Definition of terms

Metabolic abnormalities were identified using non-obese ATP III criteria [27] metabolic syndrome components (SBP/DBP > 130/85 mmHg, TG ≥ 1.7 mmol/L, HDL-C < 1.04 mmol/L in men, HDL < 1.29 mmol/L in women, FBG > 5.6 mmol/L) [28]. As for the overweight/obese participants (BMI ≥ 25.0 kg/m^2^), the presence of ≤ 1 ATP III component of metabolic syndrome were defined as MHOO, while the presence of > 1 components were defined as MUOO [20, 21].

### Data collection

BMI was calculated by height and weight (weight divided by height squared, kg/m^2^). Systolic and diastolic blood pressure (SBP, DBP) were measured using digital sphygmomanometer with a standard protocol. All blood samples were collected in the morning (7–8 a.m.) after a 10–12 h overnight fast. Serum samples (centrifugation: 3000*g* for 5 min) or whole blood samples were used as required for subsequent tests.

Serum levels of fasting blood glucose (FBG), triglycerides (TG), total cholesterol (TC), high density lipoprotein cholesterol (HDL-C), low density lipoprotein cholesterol (LDL-C), alanine aminotransferase(ALT), aspartate aminotransferase (AST), urea nitrogen (Urea), creatinine (Crea), uric acid (UA), nonesterified fatty acid (NEFA) were all examined by HITACHI7600 automatic analyzer (Hitachi Ltd, Japan). Whole blood level of HbA1c was measured by Tosoh Automated Glycohemoglobin Analyzer HLC-723 G8 (Tosoh Corporation, Japan) and several parameters of blood routine (i.e. white blood cell, neutrophil, eosnophils) were measured by Sysmex XN-20 automated hematology analyzer (Sysmex Corporation, Japan). The relevant information of the methods of above parameters see in Additional file 1: Table S1 and all indexes were measured under standardized conditions in an ISO 15189 accredited medical laboratory [29].

### IL-33, IL-5, and IL-13 measurements

Serum samples were obtained and collected into tubes and then stored at − 70 °C until use. Serum levels of IL-33 (CSB-E13000h, CUSABIO, China), IL-5 (CSB-E04636h, CUSABIO, China) and IL-13 (CSB-E04601h, CUSABIO, China) were assessed by specific commercially available enzyme-linked immunosorbent assays (ELISA). The lower limits of detection of IL-33, IL-5 and IL-13 assays were 15.6 pg/mL, 0.78 pg/mL and 62.55 pg/mL, respectively. The intra-assay coefficients of variation of IL-33, IL-5, and IL-13 assays were all 8%, and the inter-assay coefficients of variation of IL-33, IL-5, and IL-13 assays were all 10%. All of the cytokine measurements were performed simultaneously.

### Statistical analysis

Mean (SD) were given to describe the continuous variables of normal distribution and median values (and interquartile ranges (IQR)) were given to describe the continuous variables of non-normal distribution. The One-way ANOVA and Kruskal–Wallis rank sum test were used to compared the continuous variables of normal distribution and non-normal distribution among the three groups, respectively, while comparisons of nominal variables were performed using the *χ*^2^ test. The effect of gender and group on IL-33 serum level was evaluated by 2-way ANOVA. The relationship between IL-33 and clinic-metabolic parameters were analyzed by the Spearman’s correlation coefficient.

Covariates were screened based on the influence of introducing covariates into the basic model or removing covariates from the complete model, which is depended on whether the regression coefficient of IL-33 was more than 10% or the regression coefficient *p* value of covariant with respect to obesity phenotype less than 0.1. The indexes that defined the MHOO phenotype and MUOO phenotype in the present study (i.e. TG, SBP, DBP, HDL-C and FBG) were not suitable to be included into the regression model for analysis as covariates. Multivariate logistic regression analyses were performed to determine the independent correlation between IL-33 levels and metabolic phenotypes of obesity. Non-adjusted and adjusted models were used to assess confounders. In all of the models, IL-33 serum levels were treated as both continuous variables scaled to 10 pg/mL increments and categorical variables divided into tertiles. Trend analysis was then performed by modeling the IL-33 tertiles as continuous variables. Stratified analyses and interaction analyses by gender, age (≥ 45 years, < 45 years) and BMI (25–28 kg/m^2^, ≥ 28 kg/m^2^) were further conducted.

The software Empower (R) (www.empowerstats.com, X & Y Solutions Inc., Boston MA) and R (http://www.R-project.org) [30] were performed for all statistical analyses. Two-tailed *p*-values less than 0.05 were considered statistically significant.

## Results

### Clinical characteristics of the participants

Baseline characteristics of the all participants are represented in Table 1. No difference between healthy and overweight/obese subjects was observed at baseline regarding gender and age. Males made up a larger percentage in each group (65.75% of the HC group, 68.06% of the MHOO group and 66.67% of the MUOO group). With the exception of NEFA and Uric, each index showed a significant difference between three groups. In comparison with the HC subjects, both MHOO and MUOO subjects had higher BMI, SBP and DBP, higher circulating levels of TG, ALT, Crea, UA, WBC and lower HDL-C level. Compared with MHOO group, the levels of all metabolic components, TC, LDL-C, HbA1c, UA, WBC and neutrophil in MUOO group were significantly elevated.

**Table 1:** Clinical characteristics of the participants

Characteristic	HC group (n = 73)	MHOO group (n = 72)	MUOO group (n = 72)	*p*^a^
Age (years)	44.48 (12.05)	44.39 (12.74)	44.82 (12.72)	0.977
Sex				0.957
Male	48 (65.75%)	49 (68.06%)	48 (66.67%)	
Female	25 (34.25%)	23 (31.94%)	24 (33.33%)	
BMI (kg/m^2^)	21.99 (1.50)	27.38 (2.48)**	28.15 (2.71)**	1.85E−42
Metabolic components				
SBP (mmHg)	114.00 (9.38)	122.42 (12.56)**	132.85 (15.65)**^,##^	2.38E−15
DBP (mmHg)	69.38 (7.07)	77.51 (10.02)**	83.21 (10.86)**^,#^	3.02E−15
TG (mmol/L)	0.92 (0.76–1.12)	1.23 (0.90–1.57)*	2.06 (1.51–2.91)**^,##^	1.38E−11
HDL-C (mmol/L)	1.55 (0.30)	1.37 (0.23)**	1.18 (0.33)**^,##^	9.53E−12
FBG (mmol/L)	4.96 (4.72–5.26)	5.00 (4.68–5.19)	5.64 (5.07–6.12)**^,##^	1.52E−9
Biochemical measures				
TC (mmol/L)	4.54 (0.74)	4.57 (0.79)	5.06 (1.09)*^,#^	0.001
LDL-C (mmol/L)	2.53 (0.65)	2.62 (0.74)	3.01 (0.91)**^,#^	0.001
NEFA (mmol/L)	0.50 (0.20)	0.45 (0.15)	0.46 (0.09)	0.087
HbA1c (%)	5.40 (5.30–5.60)	5.40 (5.27–5.70)	5.70 (5.50–6.12)**^,##^	3.64E−7
ALT (U/L)	15.90 (11.60–21.40)	20.05 (15.38–29.48) **	25.80 (19.32–36.40)**	6.05E−7
AST (U/L)	19.20 (17.10–22.40)	20.70 (18.38–24.75)	22.55 (19.25–28.15)**	0.001
Urea (mmol/L)	5.38 (4.27–6.19)	5.31 (4.55–6.00)	5.39 (4.42–5.93)	0.873
Crea (μmol/L)	60.79 (21.88)	73.28 (17.51)*	70.41 (13.13)*	9.10E−5
UA (μmol/L)	285.10 (68.52)	319.99 (71.63)*	364.48 (70.57)**^,##^	7.31E−10
WBC (× 10^9^)	5.60 (1.53)	6.10 (1.45)*	7.23 (1.70)**^,##^	6.20E−9
Neutrophil (× 10^9^)	3.27 (1.17)	3.38 (1.13)	4.32 (1.32)**^,##^	2.56E−7
Eosnophils (× 10^9^)	0.08 (0.05–0.17)	0.12 (0.07–0.17)	0.14 (0.08–0.18) *	0.015
Cytokines				
IL-33 (pg/mL)	76.24 (21.42)	80.46 (26.65)	112.11 (36.12)**^,##^	1.83E−13
IL-5 (pg/mL)	9.33 (3.71)	10.58 (4.30)	13.28 (4.65)**^,##^	2.38E−7
IL-13 (pg/mL)	646.92 (220.21)	741.55 (291.69)*	881.93 (271.69)**^,#^	1.00E−6

Data are presented as the Mean (SD) or Median (IQR: Q1-Q3) for continuous variables and percentage for categorical variables

HC: healthy control; MHOO: metabolically healthy overweight/obese; MUOO: metabolically unhealthy overweight/obese; BMI: body mass index; SBP: systolic blood pressure; DBP: diastolic blood pressure; FBG: fasting blood glucose; TG: triglycerides; TC: total cholesterol; HDL-C: high-density lipoprotein-cholesterol; LDL-C: low-density lipoprotein-cholesterol; NEFA: non-esterified fatty acids; ALT: Alanine transaminase; AST: aspartate transaminase; Urea: blood urea nitrogen; Crea: creatinine; UA: uric acid; WBC: white blood cell

*p < 0.05, **p < 0.001 compared HC group; ^#^*p *< 0.05, ^##^*p *< 0.001 compared MHOO group

^a^ The One-way ANOVA or Kruskal–Wallis rank sum test or *χ*^2^ test were used for comparisons between subgroups

### Differences in distribution of IL-33 serum levels in three study groups

**Fig. 1:**
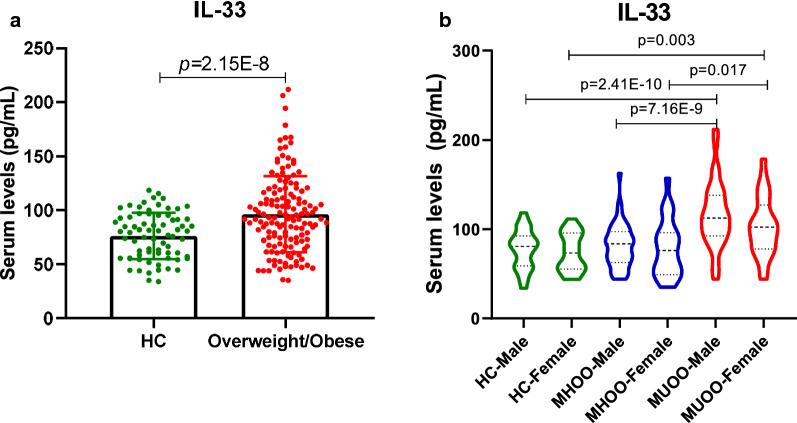
Differences in the distribution of serum IL-33 levels in each study group. **a** Box-scatter plot showing differences in IL-33 serum levels in healthy controls (n = 73) and overweight/obese subjects (*n *= 144) by box-scatter plot; Student’s t test were performed in two groups. **b** Violin plot showing basal serum IL-33 levels in each study group by sex; 2-way ANOVA analysis were performed to analyze the effect of group and sex on IL-33 level

Circulating levels of IL-33 were higher in all overweight/obese subjects than those in healthy subjects (*p* = 2.15E−8, Fig. 1a). Interestingly, serum levels of IL-33 were significantly elevated in MUOO subjects compared with MHOO subjects or HC subjects, whereas IL-33 levels in MHOO population were basically  similar to those of healthy control subjects (Table 1). IL-33 levels were also analyzed in the three groups based on gender, and the serum levels were basically the same in males and females for each group (Fig. 1b). However, IL-33 serum levels of both male (*p* = 3.41E−5) and female (*p *= 0.002) subjects in MUOO group were higher than those in the other two groups, respectively (*p* = 2.41E−10 when MUOO vs HC and *p* = 7.16E−9 when MUOO vs MHOO in male; *p *= 0.017 when MUOO vs MHOO and *p *= 0.003 when MUOO vs HC in female).

We also analyzed the serum levels of several other Th2 cytokines. Consistent with the distribution trend of IL-33 concentration, serum levels of IL-5 and IL-13 in MUOO group were also significantly higher than those in the MHOO group (Table 1).

### Association of serum IL-33 levels with metabolic phenotypes of obesity

Univariate and multivariate logistic regression analysis were performed on the association between each study group to explore the relationship between IL-33 and obesity metabolic phenotypes. In different multivariable models adjusted for potential confounders (Table 2), MUOO phenotype was significantly associated with higher serum levels of IL-33 when compared with HC participants (OR = 1.70; 95% CI 1.09–2.64; *p *= 0.019) after adjusted for all relevant confounders (model 5). However, no significant correlation was observed between MHOO phenotype and IL-33 levels. In the analyses adjusted for all relevant confounders, MUOO phenotype was still associated with higher odds of serum IL-33 levels in all overweight/obese participants (OR = 1.50; 95% CI 1.20–1.88; *p *= 2.91E−4).

**Table 2:** Multivariate analysis of the association between obesity phenotypes and serum IL-33 levels (per 10 pg/mL increment)

Models	HC vs MHOO		HC vs MUOO		MHOO vs MUOO	
	OR (95% CI)	*p* value	OR (95% CI)	*p* value	OR (95% CI)	*p* value
Model 1	1.08 (0.94–1.23)	0.293	1.59 (1.34–1.87)	1.41E−7	1.40 (1.22–1.60)	6.27E−7
Model 2	1.08 (0.94–1.23)	0.300	1.60 (1.35–1.90)	2.84E−6	1.40 (1.22–1.61)	1.79E−4
Model 3	1.06 (0.92–1.23)	0.416	1.71 (1.37–2.12)	4.23E−7	1.45 (1.24–1.71)	1.90E−7
Model 4	1.07 (0.93–1.24)	0.344	1.85 (1.39–2.46)	8.53E−5	1.48 (1.23–1.77)	5.70E−7
Model 5	0.99 (0.83–1.18)	0.942	1.70 (1.09–2.64)	0.019	1.50 (1.20–1.88)	2.91E−4

Model 1 was unadjusted; Model 2 was adjusted for sex and age; Model 3 was adjusted for model 2 plus TC, LDL-C, HbA1c and NEFA; Model 4 was adjusted for model 3 plus IL-5 and IL-13. Model 5 was adjusted for model 4 plus ALT, AST, Urea, Crea, UA, WBC, Neutrophil and Eosnophils

OR: odds ratio; CI: confidence interval

Trend  analysis also showed that MUOO phenotype rather than MHOO phenotype was positively correlated with IL-33 level (Table 3) and the ORs for MUOO phenotype were significantly higher in the third tertile of IL-33 levels than in the first tertile. Compared with HC participants, the third tertile was associated with a 13.34-fold increase in the odds of MUOO phenotype (OR = 13.34; 95% CI 1.35–132.15; *p *= 0.027) after adjusting all relevant variables (model 5). However, there was no evidence of increased risk of MHOO phenotype in all tertiles of IL-33 levels. With the MHOO participants as a reference group, the subjects with IL-33 levels in both the second (OR = 4.44; 95% CI 1.23–15.99; *p *= 0.023) and third tertiles (OR = 13.43; 95% CI 2.86–63.12; *p *= 0.001) had higher increased risk of having MUOO phenotypes after adjusting for confounders (model 5). Specifically, MHOO subjects with IL-33 levels in second tertiles had higher risk of developing MUOO compared with HC subjects under all models. In addition, multivariate logistic regression analyses were further performed to investigate the strength of the association between the circulating level of IL-33 and the susceptibility to MUOO against both HC and MHOO, and results showed MUOO phenotype was still significantly associated with higher serum levels of IL-33 when compared with both HC and MHOO participants (Additional file 2: Table S2).

**Table 3:** Multivariate analysis of the association between obesity phenotypes and serum IL-33 level tertiles

Models	HC vs MUOO		HC vs MHOO		MHOO vs MUOO	
	OR (95% CI)	*p* value	OR (95% CI)	*p* value	OR (95% CI)	*p* value
Model 1						
Tertile 2	2.28 (0.94–5.56)	0.069	1.18 (0.53–2.63)	0.683	3.09 (1.28–7.46)	0.012
Tertile 3	17.66 (6.42–48.60)	3.80E−5	1.34 (0.60–2.97)	0.477	12.78 (4.85-33.67)	1.15E−4
*p* for trend	3.89E−4		0.770		7.15E−7	
Model 2						
Tertile 2	2.36 (0.96–5.84)	0.062	1.18 (0.51–2.72)	0.706	3.20 (1.31–7.78)	0.011
Tertile 3	19.65 (6.92–55.77)	2.10E−5	1.34 (0.60–2.98)	0.479	13.87 (5.15–37.37)	1.88E−7
*p* for trend	0.007		0.979		5.29E−9	
Model 3						
Tertile 2	1.99 (0.70–5.63)	0.196	1.21 (0.51–2.85)	0.669	3.21 (1.19–8.65)	0.021
Tertile 3	25.45 (7.08-91.42)	4.76E−7	1.25 (0.54–2.87)	0.602	15.32 (4.77–49.14)	5.64E−7
*p* for trend	0.013		0.398		3.28E−5	
Model 4						
Tertile 2	2.38 (0.70–8.16)	0.166	1.19 (0.49–2.88)	0.693	2.92 (1.00–8.53)	0.050
Tertile 3	22.49 (5.36–94.34)	4.76E−7	1.31 (0.56–3.06)	0.538	13.11 (3.81–45.09)	6.30E−5
*p* for trend	0.026		0.367		1.04E−6	
Model 5						
Tertile 2	1.20 (0.17–8.73)	0.857	0.97 (0.32–2.93)	0.952	4.44 (1.23–15.99)	0.023
Tertile 3	13.34 (1.35–132.15)	0.027	0.62 (0.21–1.79)	0.376	13.43 (2.86–63.12)	0.001
*p* for trend	0.019		0.392		4.17E−4	

Model 1 was unadjusted; Model 2 was adjusted for sex and age; Model 3 was adjusted for model 2 plus TC, LDL-C, HbA1c and NEFA; Model 4 was adjusted for model 3 plus IL-5 and IL-13. Model 5 was adjusted for model 4 plus ALT, AST, Urea, Crea, UA, WBC, Neutrophil and Eosnophils

OR: odds ratio; CI: confidence interval

### Stratified and interaction analyses in overweight/obese subjects

Further stratified analysis and interaction analysis were performed by gender, age and BMI in all overweight/obese subjects (Table 4). A slight gender difference was observed in the association between IL-33 and MUOO phenotype. IL-33 level was positively correlated with MUOO in male subjects (*p* = 3.31E−4) but the correlation was not significant in female participants (*p* = 0.090). At different ages or BMIs, IL-33 levels showed positive association with MUOO. However, the interaction analysis showed that none of the above variables (i.e., gender, age and BMI) significantly changed the relationship between the IL-33 and MUOO phenotype (all *p* for the interaction > 0.05).

**Table 4:** Effect size of IL-33 on MUOO in the planned and exploratory subgroups in all overweight/obese subjects

Characteristic	No. of participants	Effect size (95% CI)	*p* value	*p* for interaction
Sex				0.188
Male	97	1.68 (1.26–2.24)	3.31E − 4	
Female	47	1.30 (0.96–1.75)	0.090	
Age (years)				0.236
< 45	71	1.43 (1.08–1.87)	3.23E − 5	
≥ 45	73	1.80 (1.27–2.55)	0.001	
BMI (kg/m^2^)				0.274
25–28	90	1.43 (1.11–1.84)	0.006	
≥ 28	54	1.87 (1.16–3.03)	0.011	

The effect size of association was quantified by OR and 95% CI. Adjusted for sex, age, TC, LDL-C, HbA1c, NEFA, ALT, AST, Urea, Crea, UA, WBC, Neutrophil and Eosnophils except the subgroup variable

CI: confidence interval

### Correlation between IL-33 serum levels and clinic-metabolic parameters in overweight/obese subjects

**Fig. 2:**
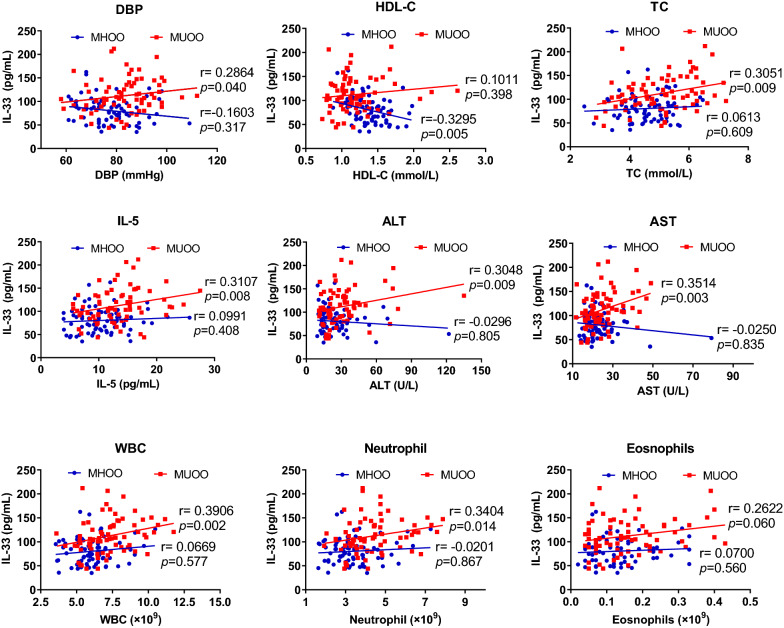
Relationship between IL-33 and major clinic-metabolic parameters in MHOO and MUOO group. Coefficients (*r*) and *P* values were calculated by the Spearman’s correlation model. The correlation level was considered significant when  < 0.05

We finally analyzed the correlation between clinic-metabolic parameters and serum levels of IL-33 in in each of the MHOO and MUOO groups separately. Our data showed that IL-33 serum levels were positively correlated with DBP, TC, ALT, AST, WBC, Neutrophil and IL-5 in MUOO subjects only in MUOO group (Fig. 2). In MHOO subjects, IL-33 had no significant association with other clinic-metabolic parameters except for negative correlated with HDL-C (Fig. 2). BMI and other biochemistry index did not significantly correlate with IL-33 in MUOO group or MHOO group.

## Discussion

Obesity, a disorder that is resulted from energy imbalance, has been considered as a chronic low-grade inflammatory disease [7, 31]. As a newly identified cytokine, IL-33 has drawn much attention in the past decade for its ability to regulate multiple immune responses and involved in the pathogenesis of various diseases [9, 11]. In the present study, our results showed that circulating levels of IL-33 in overweight/obese subjects were significantly higher than those in HC subjects, which was partly in agreement with the findings of IL-33 expression in human adipose tissue was upregulated by severe obesity [13]. Obese population with or without metabolic disorders (presented as MUOO phenotype, MHOO phenotype, respectively) could be significantly different in terms of immune and inflammatory response [7, 24, 25]. Our results confirmed that IL-33 levels were increased in MUOO subjects relative to MHOO subjects. For MHOO population, despite being overweight or obese, the circulating level of IL-33 and its distribution were basically the same as those in the healthy individuals, indicating that circulating level of IL-33 may not be significantly affected in the absence of obvious metabolic abnormalities, even if they are overweight or obese.

Although several previous studies based on obese mice models suggested that IL-33 could reduce adiposity, induce anti-inflammation effects in adipose tissue and improve glucose and lipid metabolism [13–16, 32], there is no reports on the specific correlation between circulating level of IL-33 and human obesity and its associated metabolic disorders. To this end, we analyzed the correlation between IL-33 and metabolic phenotypes of obesity and the results showed that IL-33 was significantly and positively associated with MUOO phenotype, but not associated with MHOO phenotype. Interestingly, when trend analysis was performed on the association of IL-33 with different obesity phenotypes, we found that MHOO subjects with higher IL-33 levels are more likely to develop MUOO compared to HC populations under all models. One of the possible reasons is that people with MHOO phenotype were already overweight/obese and may be more susceptible to external obesogenic/pro-inflammatory factors. What’s more, the contradiction between our results and those of previous studies indicates that IL-33 might have a more complex relationship with the progression of obesity. Several plausible explanations of the dissimilar findings are as follows.

First of all, the study models or populations are different. Our study was focused on overweight/obese population, which was different from the subjects of previous researches who are mostly severely obese people or mice. Secondly, IL-33 can be secreted by various cells or tissues in an autocrine or endocrine manner [9, 13, 14], while the physiological function or distribution pattern of IL-33 is closely related to its secretion mode and production site [33, 34]. Hence, the pathophysiological properties of IL-33 may depend on its secreting cell and temporal expression [33]. So the correlation between the IL-33 serum level and its tissue expression in different depots may be more complicated. Finally, most previous studies have shown that exogenous IL-33, rather than endogenous IL-33, can regulate glucolipid metabolism and inflammation in obesity [9, 15, 17]. Whether endogenous IL-33 can fully maintain the immune balance of obese adipose tissue is controversial [9, 14] and requires by further research.

In the current study, interaction analysis did not showed that gender, age and BMI significantly affected the association between IL-33 and MUOO phenotype, although the association between IL-33 and MUOO phenotype seems insignificant in the female population after gender stratification. We deduced that in addition to the relatively small number of female population, the menstrual cycle, sex hormone and the use of contraceptives in female population may also influence the association between IL-33 and MUOO phenotype. Further research on the mechanism of IL-33 in the occurrence and progression of obesity in different genders are also worth exploring.

In view of the significant correlation between IL-33 and MUOO phenotype, we further analyzed the relationship between IL-33 and metabolic indicators in each of the MHOO and MUOO groups and found that IL-33 was positively associated with several risk factors for cardiovascular disease only in MUOO participants. No studies have reported the relationship between IL-33 and DBP, and the results of significant positive correlation between IL-33 and TC in the MUOO population were partially consistent with those of Hasan et al. [35]. Although it is not clear why the negative correlation between IL-33 and HDL-C was only observed in MHOO population, we  deduced that the influence of elevated IL-33 on the HDL-C of cardiovascular disease may be more significant in the MHOO population, since the HDL-C level in this population was relatively higher than that in MUOO group. Therefore, our results demonstrated that IL-33 is closely related to risk factors of cardiovascular disease (e.g. blood pressure, blood lipid level). In addition to this, our study showed positive associations between IL-33 and several nonspecific inflammatory markers (i.e. ALT, AST, WBC and neutrophil) in MUOO subjects. There has been no research about the relationship between inflammatory markers and IL-33 in overweight/obese population. However, several reports have showed that IL-33 is positively correlated with transaminase activity in patients with chronic liver disease [36] and can promote the initiation and recruitment of neutrophils as a direct chemoattractant for neutrophils in inflammatory microenvironment [37, 38]. Based on the above results, our results did not support a cardio-protective effect of IL-33 in overweight/obese people, even if the correlation between IL-33 and metabolic parameters were not strong. On the contrary, the current study demonstrated that IL-33 was closely related with the metabolic-proinflammatory profile in overweight/obese individuals especially in those with MUOO phenotype.

Previous studies have shown that both Th2 and Th1 cytokines levels could be elevated in obese population as well as metabolic syndrome patients [39–41]. Given that IL-33 could regulate metabolic inflammation by engaging ILC2s and activating downstream type 2 immune response [42, 43], we tested circulating levels of Th2 cytokines IL-5/13 and found the distribution trend of IL-5/13 levels in different groups were basically consistent with that of IL-33, indicating that Th2 immune responses were activated during the progression of obesity. Thus, it was not difficult to infer that both pro-inflammatory and anti-inflammatory responses were induced in overweight/obese population with metabolic disorders. A recent study has found that adipose derived IL-33 was upregulated in response to short-term high-fat diet, while promoting Th2 immune response and inducing anti-inflammatory response to maintain tissue homeostasis [6], suggesting that adipose tissue could balance local inflammatory responses through a protective feedback mechanism mediated by IL-33.

Although the biological role of IL-33 on MUOO phenotype was not explored in this study, our data could provide an important hint that increased circulating IL-33 level in overweight/obese people with metabolic disorders might be involved in a protective feedback mechanism of immune imbalance caused by obesity and its related metabolic disorders. In any case, our results suggested that the notion that IL-33 was always beneficial in the metabolic diseases was naïve and provide a validation for an association between IL-33 serum level and obesity metabolic phenotypes in Chinese population.

Several limitations are worth consideration when interpreting our findings. First, the criteria for MHOO are not uniform worldwide [21]; thus, part of the results in our study may be affected to some extent by the difference in MHOO definition. Second, information about the daily alcohol intake, dietary intake, work environment, data on the body fat distribution of the participants, sex hormone levels and menstrual cycle of the female population was not effectively collected, which may have influenced the results of this study to some extent. Third, this research has yet to test insulin level and Th1 cytokine levels of the participants, so it was not possible to assess the presence of insulin resistance, which is a major cause of obesity and its related metabolic diseases. Fourth, the IL-33 expression in adipose tissues of the participants was unable to detect in this study; Future research on the role of IL-33 in metabolic diseases from the perspectives of comparing tissue levels and circulatory levels will help to further reveal its action on metabolic disease. Additionally, since the temporal relationship between serum level of IL-33 and the occurrence of metabolic disorders in overweight/obese population was not known, we cannot conclude that IL-33 directly causes metabolic disorders in overweight/obese patients. Prospective longitudinal studies are needed to investigate the causal relationship of IL-33 level and development of obesity.

## Conclusions

Circulating levels of IL-33 in overweight/obese people with various metabolic phenotypes were different and was significantly elevated only in MUOO population; IL-33 levels were positively correlated with MUOO phenotype and metabolic syndrome risk factors in overweight/obese Chinese adults.

## Supplementary information

**Additional file 1:** Table S1. Methods for the determination of the studied biochemical parameters.

**Additional file 2: **Table S2. Multivariate analysis of the association between IL-33 levels and susceptibility to MUOO against both HC and MHOO.

## Data Availability

The datasets used and/or analysed during the current study are available from the corresponding author on reasonable request.

## Abbreviations

HC: Healthy control
MHOO: Metabolically healthy overweight/obese
MUOO: Metabolically unhealthy overweight/obese
BMI: Body mass index
T2DM: Type 2 diabetes mellitus
SBP: Systolic blood pressure
DBP: Diastolic blood pressure
FBG: Fasting blood glucose
TG: Triglycerides
TC: Total cholesterol
HDL-C: High-density lipoprotein-cholesterol
LDL-C: Low-density lipoprotein-cholesterol
NEFA: Non-esterified fatty acids
ALT: Alanine transaminase
AST: Aspartate transaminase
Urea: Blood urea nitrogen
Crea: Creatinine
UA: Uric acid
WBC: White blood cell
IQR: Interquartile range
OR: Odd ratio
CI: Confidence interval
