# Supplementary material for: Circulating levels of IL-33 are elevated by obesity and positively correlated with metabolic disorders in Chinese adults
Source: J Transl Med. 2021 Feb 4;19:52. doi: 10.1186/s12967-021-02711-x (PMC7863234; doi:10.1186/s12967-021-02711-x)
Supplement: Supplementary file 1 — Additional file 1: Table S1. Methods for the determination of the studied biochemical parameters. [file 12967_2021_2711_MOESM1_ESM.docx]

**Table S1. Methods for the determination of the studied biochemical parameters**

| **Parameters** | **Method** | **%CV** | **Instruments** | **Manufacturer** |
| --- | --- | --- | --- | --- |
| FBG | hexokinase method | 2% | HITACHI7600 automatic analyzer | Hitachi Ltd |
| TG | glycerol phosphate oxidase-PAP method | 5% | HITACHI7600 automatic analyzer | Hitachi Ltd |
| TC | Cholesterol oxidase method | 5% | HITACHI7600 automatic analyzer | Hitachi Ltd |
| HDL-C | selective inhibition method | 5% | HITACHI7600 automatic analyzer | Hitachi Ltd |
| LDL-C | selective elimination method | 5% | HITACHI7600 automatic analyzer | Hitachi Ltd |
| NEFA | Enzymatic method | 10% | HITACHI7600 automatic analyzer | Hitachi Ltd |
| HbA1c | HPLC | 5% | Tosoh Automated Glycohemoglobin Analyzer HLC-723 G8 | Tosoh Corporation |
| ALT | Enzymatic method | 10% | HITACHI7600 automatic analyzer | Hitachi Ltd |
| AST | Enzymatic method | 10% | HITACHI7600 automatic analyzer | Hitachi Ltd |
| Urea | urease method | 3% | HITACHI7600 automatic analyzer | Hitachi Ltd |
| UA | Enzymatic method | 5% | HITACHI7600 automatic analyzer | Hitachi Ltd |
| Crea | Enzymatic method | 5% | HITACHI7600 automatic analyzer | Hitachi Ltd |
| WBC | Flow cytometry | 4% | Sysmex XN-20 automated hematology analyzer | Sysmex Corporation |
| neutrophil | Flow cytometry | 4% | Sysmex XN-20 automated hematology analyzer | Sysmex Corporation |
| eosnophils | Flow cytometry | 4% | Sysmex XN-20 automated hematology analyzer | Sysmex Corporation |

**Abbreviations**: CV: coefficients of variation; FBG: fasting blood glucose, TG: triglycerides, TC: total cholesterol, HDL-C: high-density lipoprotein-cholesterol, LDL-C: low-density lipoprotein-cholesterol, NEFA: non-esterified fatty acids. ALT: Alanine transaminase; AST: aspartate transaminase, Urea: blood urea nitrogen, Crea: creatinine, UA: uric acid, WBC: white blood cell; HPLC: High performance liquid chromatography.
