# Supplementary material for: Circulating levels of IL-33 are elevated by obesity and positively correlated with metabolic disorders in Chinese adults
Source: J Transl Med. 2021 Feb 4;19:52. doi: 10.1186/s12967-021-02711-x (PMC7863234; doi:10.1186/s12967-021-02711-x)
Supplement: Supplementary file 2 — Additional file 2: Table S2. Multivariate analysis of the association between IL-33 levels and susceptibility to MUOO against both HC and MHOO. [file 12967_2021_2711_MOESM2_ESM.docx]

**Table S2. Multivariate analysis of the association between IL-33 levels and susceptibility to MUOO against both HC and MHOO**

| **Exposure** | **Model 1** | | **Model 2** | | **Model 3** | | **Model 4** | |
| --- | --- | --- | --- | --- | --- | --- | --- | --- |
|  | **OR (95%CI)** | ***p*** | **OR (95%CI)** | ***p*** | **OR (95%CI)** | ***p*** | **OR (95%CI)** | ***p*** |
| **IL-33 levels** | | | | | | | | |
| **Per 10pg/mL increment** | 1.49  (1.31-1.70) | 1.95E-8 | 1.50  (1.31-1.70) | 1.04E-5 | 1.51  (1.30-1.76) | 6.91E-7 | 1.49  (1.23-1.82) | 2.42E-6 |
| Tertile 1 | 1.0 |  | 1.0 |  | 1.0 |  | 1.0 |  |
| Tertile 2 | 2.87  (1.16-7.07) | 0.022 | 2.89  (1.17-7.15) | 0.022 | 2.85  (1.04-7.84) | 0.042 | 1.83  (0.50-6.65) | 0.263 |
| Tertile 3 | 12.86  (5.37-30.79) | 5.29E-6 | 13.33  (5.52-32.21) | 1.42E-7 | 15.65  (5.59-43.84) | 5.41E-5 | 14.08  (3.55-55.77) | 7.72E-5 |
| ***p* trend** | 4.52E-7 | | 3.79E-6 | | 7.13E-4 | | 2.83E-5 | |

**Notes:** Model 1 was unadjusted; Model 2 was adjusted for sex and age; Model 3 was adjusted for model 2 plus TC, LDL-C, HbA1c and NEFA; Model 4 was adjusted for model 3 plus IL-5, IL-13, ALT, AST, Urea, Crea, UA, WBC, Neutrophil and Eosnophils.

**Abbreviations**: OR: odds ratio, CI: confidence interval.
